# Ambulance smartphone tool for field triage of ruptured aortic aneurysms (FILTR): study protocol for a prospective observational validation of diagnostic accuracy

**DOI:** 10.1136/bmjopen-2016-011308

**Published:** 2016-10-24

**Authors:** Thomas L Lewis, Rachael T Fothergill, Aneurysm-FILTR Study Group, Alan Karthikesalingam

**Affiliations:** 1 St George's Vascular Institute, St George's University of London, London, UK; 2 Clinical Audit & Research Unit, London Ambulance Service NHS Trust, 8-20 Pocock Street, London, UK; 3 Cardiovascular and Cell Sciences Institute, St George's University of London, London, UK

**Keywords:** smartphone, mobile app, pre-hospital, ruptured abdominal aortic aneurysm, VASCULAR SURGERY

## Abstract

**Introduction:**

Rupture of an abdominal aortic aneurysm (rAAA) carries a considerable mortality rate and is often fatal. rAAA can be treated through open or endovascular surgical intervention and it is possible that more rapid access to definitive intervention might be a key aspect of improving mortality for rAAA. Diagnosis is not always straightforward with up to 42% of rAAA initially misdiagnosed, introducing potentially harmful delay. There is a need for an effective clinical decision support tool for accurate prehospital diagnosis and triage to enable transfer to an appropriate centre.

**Methods and analysis:**

Prospective multicentre observational study assessing the diagnostic accuracy of a prehospital smartphone triage tool for detection of rAAA. The study will be conducted across London in conjunction with London Ambulance Service (LAS). A logistic score predicting the risk of rAAA by assessing ten key parameters was developed and retrospectively validated through logistic regression analysis of ambulance records and Hospital Episode Statistics data for 2200 patients from 2005 to 2010. The triage tool is integrated into a secure mobile app for major smartphone platforms. Key parameters collected from the app will be retrospectively matched with final hospital discharge diagnosis for each patient encounter. The primary outcome is to assess the sensitivity, specificity and positive predictive value of the rAAA triage tool logistic score in prospective use as a mob app for prehospital ambulance clinicians. Data collection started in November 2014 and the study will recruit a minimum of 1150 non-consecutive patients over a time period of 2 years.

**Ethics and dissemination:**

Full ethical approval has been gained for this study. The results of this study will be disseminated in peer-reviewed publications, and international/national presentations.

**Trial registration number:**

CPMS 16459; pre-results.

**Strengths and limitations of this study**

- Prospective multicentre observational study across major city.
- Large number of patient cases in target population.
- Use of mobile technology to facilitate accurate data collection.
- Assessment and validation of clinical decision-making tool for prehospital diagnosis of ruptured abdominal aortic aneurysm.
- Potential for non-random patient selection and confounding introducing bias.
- Reliant on accurate documentation of final hospital discharge diagnosis for each patient encounter.
- Incomplete data entry sets are not saved.

## Background

Abdominal aortic aneurysms (AAAs) are a significant health problem that account for 4 deaths per 100 000 population per year.^1^ Rupture of an abdominal aortic aneurysm (rAAA) carries a high mortality rate and is often fatal.^2^ ^3^ Although rare cases survive conservative management following ‘contained rupture’, survival from rAAA is generally only possible with definitive surgery, which can be either open repair or endovascular surgical intervention (EVAR). For patients with rAAA, many non-operative factors are key determinants of survival.^4^ The lowest mortality for rAAA is seen in hospitals that have greater bed capacities, greater availability of support services, higher annual procedural caseload of AAA repair with a greater proportion of cases undergoing endovascular repair.^4^ Data from a large prospective multicentre randomised clinical trial of different surgical techniques (IMPROVE) suggests that the type of surgery itself may not play as great a role in overall survival.^2^ ^5^ ^6^ In-hospital survival from rAAA, intervention rates and uptake of endovascular repair are lower in England than in the USA, further suggesting there is room for improvement in the organisation and delivery of care.

It is possible that more rapid access to specialist care might improve mortality for rAAA, though selection of the fittest patients for transfer precludes definitive evidence to support this likelihood.^7–11^ Currently in London, patients are taken to the nearest hospital regardless of its ability to provide repair for rAAA,^12^ and protocols for triage of suspected rAAA are not routine across the UK. Diagnosis is not always straightforward with up to 42% of rAAA initially misdiagnosed, introducing potentially harmful delay when patients require interhospital transfer.^13^ The signs and symptoms of rAAA may be unreliable and patients frequently show additional features that may confound the diagnosis.^14^ There is a need for an effective clinical decision support tool for accurate prehospital diagnosis and triage to enable transfer to an appropriate centre. Such triage systems are already in place for patients with suspected stroke, ST elevation myocardial infarction (STEMI) and major trauma which have all been shown to be effective in reducing morbidity and mortality.^15–17^ In London, patients with suspected rAAA are currently taken to the nearest hospital for further assessment, regardless of its capability to repair rAAA. This can introduce additional delays for patients who subsequently require transfer to a vascular service, which in turn might increase mortality. It is possible that rapid more accurate diagnostic assessment in the prehospital environment and direct transfer to a vascular centre capable of providing definitive intervention might permit reduced mortality for populations with rAAA. However, there are currently no clinical triage tools to act as decision aids for prehospital service providers to estimate the risk of rAAA diagnosis based on presenting symptoms and medical assessment. If rAAA could be more accurately diagnosed in the prehospital setting, there is potential that more patients might receive definitive care. Any such tool should be accurate and feasible for use in the prehospital setting.

FILTR is a pilot prospective multicentre observational study, assessing the diagnostic accuracy of an ambulance smartphone triage tool for detection of rAAA. The first stage in developing a prehospital triage system requires validation and assessment of a clinical decision support system. This will be conducted across London in conjunction with London Ambulance Service (LAS). LAS clinicians will use a logistic score developed at St George's Vascular Institute for use as a mobile app on a smartphone platform. During each patient encounter, key parameters required to predict risk of rAAA will be collected using the app. Final discharge diagnosis will be retrospectively identified and collated for each encounter. This will enable assessment of the feasibility, validity and safety of the prehospital smartphone triage tool for rAAA. Secondary analysis will also allow modelling of the implications of such an approach for ‘false positive’ patients with other differential diagnoses if transferred to a vascular centre, or the implications of such an approach for service configuration and workload.

## Methods

### Trial design and objectives

FILTR is a prospective multicentre observational study assessing the diagnostic accuracy of an ambulance smartphone triage tool for detection of rAAA for 2 years. The objective of FILTR is to assess whether a logistic score hosted on a smartphone app can be feasible and accurate for diagnosis of rAAA at the scene of an emergency.

### Ethical approval and dissemination

Full ethical approval has been gained from the NRES Committee North West, reference 14/NW/0123. Further consultation and approval has been obtained from the NHS Health Research Authority Confidentiality Advisory Group, reference: 15/CAG/0152. The results of this study will be disseminated in peer-reviewed publications and international/national presentations.

### Funding

FILTR is supported by an Academy of Medical Sciences Clinical Lecturer Starter Grant awarded to author AK. It is also supported by grants from the Laerdal Foundation and South West London Academic Health and Social Care System.

### Registration

This is a portfolio study with UK Clinical Research Network Study Portfolio no. UKCRN ID 16459.

### Inclusion criteria

All patients over the age of 18 years assessed by a LAS clinician with acute abdominal/back/chest pain or collapse requiring transfer to hospital who do not fulfil entry criteria for existing triage tools such as myocardial infarction (MI), stroke and major trauma during the recruitment period will be eligible for inclusion.

### Exclusion criteria

The exclusion criteria are:

1. Any patient under the age of 18 years
2. Any patient not requiring transfer to hospital
3. Any patient with confirmed or suspected acute myocardial infarction (MI), major trauma or stroke as existing triage tools already exist for this condition

### Smartphone triage tool

St George's Vascular Institute and the London Ambulance Service have developed a scoring system to diagnose rAAA based on ten key parameters. The score was developed with logistic regression of ambulance records and Hospital Episode Statistics (HES) data for 2200 patients from 2005 to 2010. The score used routine ambulance clinician assessments and detected rAAA with 82.6% sensitivity (area under the receiver operating characteristic (ROC) curve 0.86). The parameters collected by the app are: age, gender, blood pressure, capillary refill time, temperature, and history of collapse, back pain, diabetes, cancer or peripheral vascular disease. This triage tool has been integrated into a secure mobile app available for smartphones on all major operating systems. This mobile app has been made available to all LAS clinicians for the observational study since November 2014. LAS clinicians, including paramedics, paramedic students and technicians, will download the app onto their own personal smartphone. A website was set up to coordinate educational meetings and provide regular study updates.^18^ LAS clinicians are advised to enter physiological parameter data as soon as it is safe to do so from a clinical perspective using the first available set of observations, and in the same circumstances as completing existing patient report forms (PRFs). Pilot results show that data entry takes an average (mean) of 16 s per patient. There will be no change to the underlying logistic regression model during the course of the study, and no intention to update or modify the app thereby preventing issues with version control. The app design prevents the saving of incomplete data entry at the point of care. Incomplete patient records will be included in the analysis, where possible as per the Strengthening the Reporting of Observational Studies in Epidemiology (STROBE) guidelines. There is the possibility for incomplete data in terms of diagnosis retrieval, outcome (mortality) retrieval and discordance between the PRF data and the app data. Owing to the study design, we will not be able to follow-up eligible patients whose data are not originally included in the app. This should not prove a limitation of the study as the aim of the study is to assess the sensitivity and specificity of the model for prediction of rAAA. Assessment of any incomplete data entries will provide an outcome measure regarding potential app user error rate. This will include a sensitivity analysis whereby the ROC curve for mortality and rAAA diagnosis using each combination of ‘app data’, ‘PRF data’ and ‘app/PRF concordant data’ will be produced.

### Primary outcome

1. To assess and validate the sensitivity and specificity of the rAAA smartphone triage tool

### Secondary outcomes

1. To assess feasibility and safety of a mobile app-based clinical decision support system delivered at the point of care in a pre-hospital setting. This includes cost-effectiveness, research effectiveness, paramedic satisfaction and overall utility.
2. To model the financial effect and workload for LAS, emergency departments and tertiary vascular centres if the destination of a patient with suspected rAAA was determined by the smartphone triage tool, for example, the extra distance/time required for the ambulance to travel to a specialist hospital; or the modelled outcomes of care for ‘false positive’ patients without rAAA transferred for care at a vascular centre.

### Power calculation

Modelling from HES data covering the period 2005–2010 estimates that over a 12-month period 150 rAAA and over 1000 controls (ie, patients found not to have a rAAA) will be studied. The study will recruit a minimum of 1150 non-consecutive patients. Expected CIs associated with predicted model performance are as follows: 83% sensitivity (95% CI 75.9% to 88.2%); 73% specificity (95% CI 69.0% to 78.3%). The expected ROC curve characteristic will have an area under the curve statistic of 0.85±0.01.

### Statistical analysis

True positive and true negative diagnoses will be defined according to the hospital discharge record retrieved by site investigators at each participating hospital. Contingency table analysis will allow reporting of sensitivity, specificity, positive predictive and negative predictive rates for the logistic score, with CIs determined by sample size. The development and validation data sets will be further explored for model refinement using a logistic regression approach, with the same entry variables entered into forward selection, removal from models at p<0.1, and significance ascribed at p<0.05. An ROC curve will be reported for rAAA diagnosis using the original logistic score, and the refined score at the study end. Separate logistic models will be designed to explore 30-day mortality. Where diagnosis or mortality data cannot be retrieved, ROC analysis is impossible. As per STROBE guidelines, this study will fully describe the available data for patients with diagnoses/mortality and those without diagnosis/mortality, and subject the demographics/presenting features of both cohorts to statistical comparison to explore whether the absence of diagnosis data has introduced selection bias to the study.

### Patient pathway

**Figure 1:**
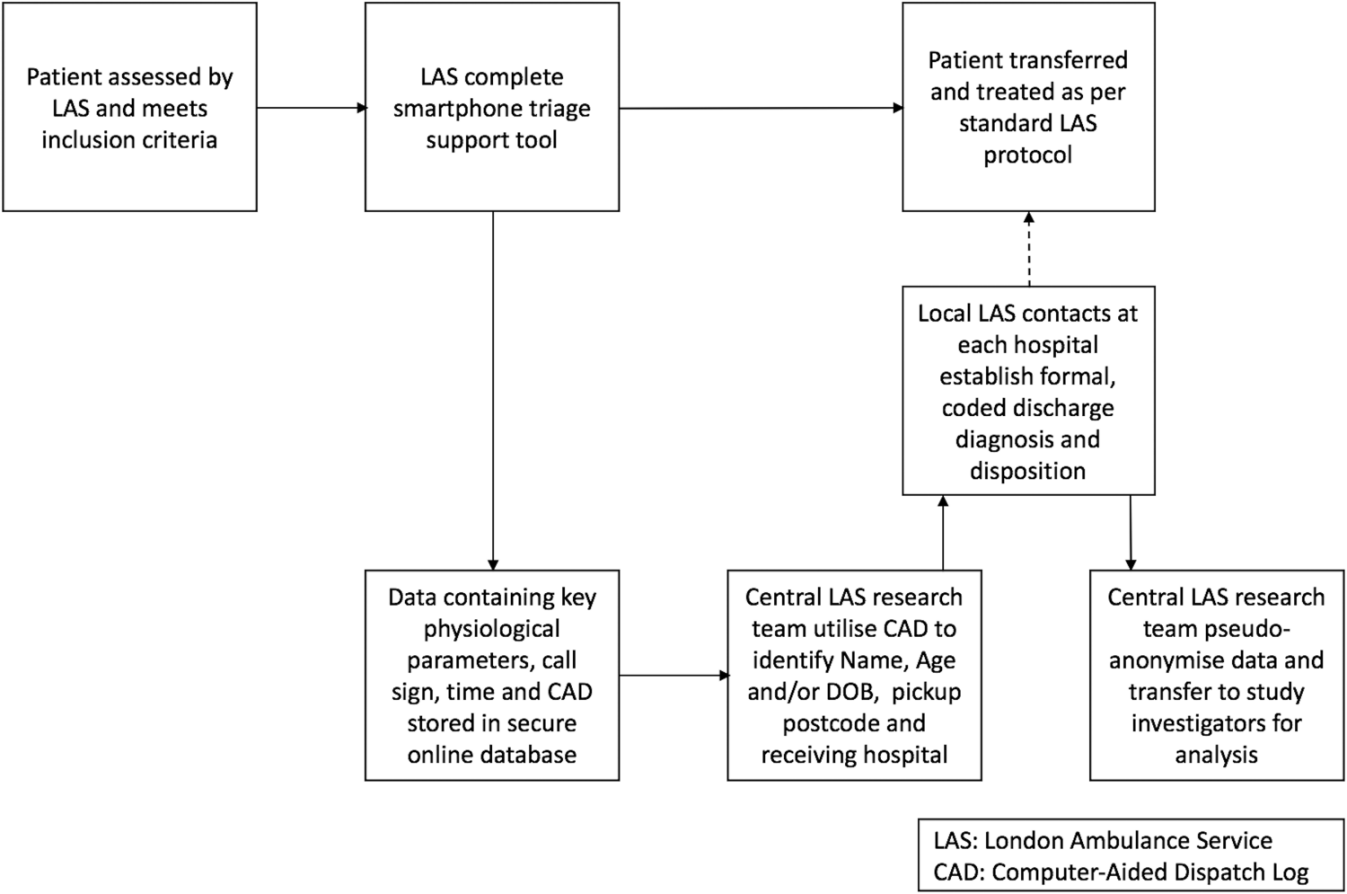
Flow diagram indicating patient and data pathway for FILTR prospective multicentre observational study. CAD, computer-aided dispatch log; DOB, date of birth; LAS, London Ambulance Service.

The patient pathway and data collection is outlined in figure 1. The data collection process uses a pre-existing service evaluation framework currently in use by LAS to identify in-hospital discharge diagnosis for patients using their service. At no point does this study alter patient triage, treatment or destination. A secure website was set up to collect and store the logistic score from the app.

### Informed consent

LAS clinicians, in line with General Medical Council (GMC) guidance regarding consent in emergencies, will not need to seek formal consent from patients in order to enrol them in this observational study. This is determined on the basis that patients with suspected rAAA may be in extremis and therefore treatment provided is immediately necessary to ‘save their life to prevent serious deterioration of their condition’ (GMC Guidance on Consent). This has been discussed with the NHS Health Research Authority, who agree that this is appropriate given the nature of the study. No patient-identifiable data are collected during the course of medical assessment and transfer to definitive care.

### Treatment and follow-up

This observational study will have no impact on triage, treatment or follow-up for the patients involved.

## Discussion

In-hospital mortality for rAAA who survive to the point of hospital admission is estimated to be 65.9% for England,^19^ with lower mortality achieved in the group selected for operative management, where 1-year mortality is 38.6% following EVAR and 42.8% following open repair.^3^ There remains ongoing uncertainty regarding how to reduce mortality with many non-operative factors being key determinants of survival. Current recommendations suggest that reductions in mortality might be achieved by rapid direct transfer to a high-volume specialist centre, that offers ready availability of the full spectrum of endovascular and open surgical care, with appropriate support facility and intensive care. This is at odds with existing prehospital service provision for patients with suspected rAAA, who are usually taken to the nearest hospital, often requiring secondary interhospital transfer for definitive care. Prehospital triage systems have been shown to be successful for other conditions such as major trauma, STEMI and stroke, and there are possibly significant implications if a similar system could be implemented for rAAA.^15–17^ Challenges for such an approach will include the diagnostic accuracy with which rAAA can be triaged at the scene of the emergency, and also the fate of patients incorrectly diagnosed with rAAA and nonetheless subjected to prolonged transfer and management at a vascular centre. Finally, outcomes in patients with rAAA that were not identified will also require investigation and estimation.

Given this study links prehospital and hospital care, an extensive collaborative approach has been employed to the benefit of the study design and patient pathway. In order to be pragmatic, existing information pathways for data collection have been used and the smartphone app records only routinely collected data. At no point does this study alter patient triage, treatment or destination, but it may provide support for an efficacy study of field triage in the future.

This prospective validation study uses an observational approach to test the diagnostic accuracy of a smartphone triage tool to support clinical decision-making for the diagnosis of rAAA at the scene of the emergency. We anticipate that assessment and validation of the smartphone triage tool will provide feasibility evidence for the use of smartphones in the emergency setting and their use in a prospective observational study; as well as provide data for whether such an approach could be beneficial to patients with rAAA without unacceptable consequences for the wider population with differential diagnoses suspected to be rAAA. The study is potentially limited by geographical issues, such as generalisability beyond London to less urban healthcare settings. It will not be possible to collect data for patients eligible but not recruited in FILTR, and the reliability with which paramedics use each aspect of the app will not be monitored prospectively, though retrospective audit will be performed between LAS paper records and electronic FILTR records for each included patient. It is not possible to identify which data fields are most difficult to enter due to the app not saving incomplete data entry sets.

### Status

The study started recruitment in November 2014 and will close recruitment in November 2016.
